# Personal Values and Psychological Well-Being Among Emerging Adults: The Mediating Role of Meaning in Life

**DOI:** 10.3390/brainsci15090930

**Published:** 2025-08-27

**Authors:** Marianna Chmiel, Zdzisław Kroplewski

**Affiliations:** Institute of Psychology, University of Szczecin, 71-017 Szczecin, Poland; zdzislaw.kroplewski@usz.edu.pl

**Keywords:** psychological well-being, personal values, meaning in life, emerging adults

## Abstract

Background/Objectives: Emerging adulthood involves identity exploration, instability, and a sense of being “in-between” adolescence and full adulthood. This study examined whether growth-oriented values (openness to change and self-transcendence) are associated with psychological well-being among emerging adults, and whether meaning in life (presence and search) is related to these variables. Methods: The study included 200 participants (M = 21.90, SD = 2.48). The following measures were used: the Psychological Well-Being Scales, the Meaning in Life Questionnaire, and the Portrait Values Questionnaire. Correlation and multiple regression analyses were conducted. Results: All key variables (psychological well-being, presence of meaning, search for meaning, openness to change, and self-transcendence) were significantly positively correlated (r = 0.27–0.74, *p* < 0.01). The presence of meaning explained the associations between both openness to change (β = 0.22, 95% CI [0.50, 1.26]) and self-transcendence (β = 0.20, 95% CI [0.36, 0.91]) with psychological well-being, whereas the search for meaning was not a significant intervening variable in either model. Conclusions: These findings highlight the relevance of growth-oriented values and the presence of meaning in understanding psychological well-being among emerging adults. Longitudinal research is needed to clarify the directionality of these relationships.

## 1. Introduction

Contemporary demographic and social changes, such as prolonged participation in education, delayed entry into marriage, and postponement of parenthood, have contributed to the emergence of a distinct developmental period situated between adolescence and full adulthood, referred to as *emerging adulthood* [1]. Initially conceptualized by Arnett as spanning ages 18 to 25 [2], this life stage has since been extended to encompass individuals up to age 29 [3]. Arnett emphasized that this phase is not merely a continuation of adolescence or an early form of adulthood, but a distinct developmental period, both theoretically and empirically.

One of the key reasons Arnett proposed the new term “emerging adulthood” rather than “early adulthood” was the observation that many individuals in this age group do not yet consider themselves full adults. Instead, they experience this time as a transitional phase (marked by exploration and uncertainty) on the path to adulthood [2,4]. This period is characterized by a relative independence from traditional social roles and normative expectations. Emerging adults often find themselves in a liminal state between full dependence and autonomy, navigating new freedoms while lacking the permanent commitments typically associated with adulthood [4].

Arnett identified five core features of emerging adulthood: identity exploration, instability, self-focus, a feeling of being in-between, and a sense of possibilities [4]. These characteristics manifest across various life domains—career, relationships, personal values—and reflect both the openness and psychological vulnerability of this life stage. Economic, cultural, and societal transformations have further contributed to the extension of this period. For example, increased access to higher education, unstable labor markets, and shifting expectations regarding marriage and parenthood have delayed traditional adult milestones for many young people [1,4]. The frequent changes in relationships, education, and employment, as highlighted by Arnett [4], may lead to a sense of uncertainty and ambivalence. While Arnett does not explicitly discuss this, the psychological demands of navigating such instability imply a need for internal psychological resources to successfully manage this stage.

In this context, the issue of psychological well-being becomes particularly relevant—not merely as the absence of psychopathology, but as positive functioning across multiple domains of life, consistent with the eudaimonic framework proposed by Ryff [5]. The pursuit of psychological well-being may be closely tied to an individual’s value system—a cognitive–affective structure that underlies decisions, motivation, and life goals, as conceptualized in Schwartz’s theory of basic human values [6]. Alongside the process of forming and clarifying values, a salient developmental task is the search for meaning in life—the need for coherence, direction, and existential significance. According to the model developed by Steger and colleagues [7], meaning in life is a multidimensional construct, encompassing both the presence of meaning and the active search for it. This article explores the associations between personal values, meaning in life, and psychological well-being, with particular attention to the potential indirect role of the presence of meaning and search for meaning. It consists of five main sections: Section 1 outlines the main constructs examined in the study and discusses the theoretical background; Section 2 details the sample characteristics, measurement instruments, procedure, and methods of data analysis; Section 3 reports the outcomes of the conducted statistical tests; Section 4 analyzes the results in the context of the proposed hypotheses and relevant theories; and Section 5 concludes with a synthesis of the most important findings from the study.

### 1.1. Psychological Well-Being and Personal Values

The term *well-being* was first introduced in the World Health Organization’s 1948 definition of health, which described it as “a state of complete physical, mental and social well-being and not merely the absence of disease or infirmity” [8] (p. 1). Although this definition referred primarily to overall health and quality of life, it marked an important shift toward a more holistic understanding of human functioning. In psychological science, well-being has since evolved into a distinct construct, examined independently of physical health, with an emphasis on optimal mental functioning and personal fulfillment across various life domains.

In the contemporary psychological literature, two primary conceptualizations of well-being are distinguished: the hedonic and the eudaimonic. The hedonic approach, associated with subjective well-being, emphasizes life satisfaction and the prevalence of positive over negative affect [9]. The eudaimonic perspective, rooted in Aristotelian philosophy, instead stresses personal growth, self-realization, and living in accordance with one’s core values [5].

Among the most widely recognized eudaimonic models is Ryff’s multidimensional conceptualization of psychological well-being [5]. Ryff frames well-being as a central component of healthy development and adopts a holistic perspective that contrasts with the affect-centered focus of hedonic theories [10]. In this model, psychological well-being is understood as the positive psychological functioning of the individual, encompassing six core dimensions: *self-acceptance* (a positive attitude toward oneself and one’s past life), *personal growth* (a feeling of continued development and the realization of one’s potential), *purpose in life* (having goals and a sense of directedness that give life meaning), *autonomy* (self-determination and independence in thought and action), *environmental mastery* (the capacity to manage one’s life and surrounding world effectively), and *positive relations* with others (having warm, satisfying, and trusting interpersonal relationships) [5].

The dimensions of psychological well-being proposed by Ryff [5] can be meaningfully related to key developmental tasks characteristic of emerging adulthood, as conceptualized by Arnett. Self-acceptance, personal growth, and purpose in life align with the process of identity explorations, understood as seeking one’s own values and direction in the domains of relationships, work, and worldviews [1]. Arnett also addresses autonomy in relation to self-focus, defined as an increased orientation toward one’s own needs and the development of independence [2]. Finally, environmental mastery and positive relations with others may reflect objective indicators of attaining adult status, such as achieving financial self-sufficiency and forming committed romantic partnerships [1].

Since the exploration of values is a key component of identity development during emerging adulthood [2], Schwartz’s theory of basic human values [6] offers a meaningful framework for understanding the motivational structure underlying this process. According to Schwartz [6], basic human values are defined as trans-situational goals of varying importance that serve as guiding principles in the life of an individual or group. They are organized into a coherent system that underlies individual decision-making processes, attitudes, and behaviors, and may help explain how individuals interpret and respond to life circumstances [6,11].

This system is structured in the form of a circular continuum, as proposed by Schwartz, where ten broad values are arranged in the original model [6], and nineteen in the refined version [11]. Each value is grounded in one or more of three universal requirements of human existence: biological needs of the organism, requirements of coordinated social interaction, and the demands placed on groups for survival and well-being [12].

In the refined value model, values are positioned along two overarching motivational dimensions: growth versus self-protection and social focus versus personal focus. Based on these dimensions, the model also groups values into four higher-order categories: *openness to change*, which emphasizes personal growth, novelty-seeking, and openness to innovation and diversity; *self-enhancement*, which focuses on personal success, power, and the pursuit of achievement-related satisfaction; *conservation*, which is concerned with maintaining stability, preserving traditions and social norms, and ensuring personal and societal security; and *self-transcendence*, which involves concern for the welfare of others, social justice, and actions oriented toward the common good and sustainable development [11]. Importantly, certain values: hedonism, face, and humility, are considered borderline values, meaning they share motivational content with two adjacent higher-order categories. In the present study, we follow the classification proposed by Schwartz and colleagues [13], according to which hedonism is included in openness to change, face in self-enhancement, and humility in conservation. The 19 values from Schwartz’s refined theory of basic values [11], grouped into four higher-order value groups, are presented in Table 1.

This structural and motivational organization of values provides a theoretical basis for investigating their relevance to psychological functioning. Values related to self-growth, such as self-transcendence and openness to change, emphasize intrinsic goals, authenticity, and the pursuit of self-development. These motivational orientations conceptually overlap with the six dimensions of psychological well-being proposed by Ryff [5], particularly those reflecting growth-related functioning, such as personal growth, purpose in life, autonomy, and positive relations with others. As Schwartz and colleagues [11] note, growth-oriented values reflect motivations for expansion and self-realization, and are more likely to guide behavior in the absence of anxiety. Empirical findings are consistent with this theoretical perspective: Bojanowska and Czerw [14] found that endorsing growth values is positively associated with higher levels of eudaimonic well-being, suggesting that individuals who prioritize these values are more likely to experience a deeper sense of fulfillment and psychological functioning consistent with the eudaimonic model.

### 1.2. Potential Statistical Mediating Role of the Presence of Meaning and Search for Meaning in Life

Emerging adulthood, as defined by Arnett [2,4], is a developmental period characterized by intensified exploration of identity, personal values, and long-term life direction. Within this life stage, the question of meaning in life often becomes particularly salient, as individuals seek to make sense of their experiences and construct a coherent narrative about who they are and who they wish to become. To better understand this process, it is useful to consider a psychological definition of meaning in life. According to Steger [15], *meaning in life* refers to the extent to which individuals understand their existence, perceive it as coherent, and regard it as significant. It also encompasses the belief that life is guided by a purpose, mission, or overarching direction that lends value to one’s actions.

Steger and colleagues [16] highlighted the essential role of time in the experience of meaning. They argued that it is important not only to experience a sense of meaning and purpose in the present, but also to actively engage in its pursuit in the future. The search for meaning is described as a dynamic, effortful process that requires persistence and commitment in order to better understand one’s life and its significance. These two temporal perspectives, experiencing meaning and seeking meaning, serve distinct but complementary functions in an individual’s development.

The presence of meaning refers to the degree to which individuals perceive their lives as significant, purposeful, and meaningful. It reflects a sense of clarity about one’s self, goals, values, and place in the world. In contrast, the search for meaning describes the degree of active striving to find or deepen one’s sense of purpose and meaning in life. It involves cognitive and emotional engagement in the process of exploring new sources of meaning and understanding one’s existence more fully [7]. Both dimensions have been shown to relate meaningfully to broader motivational systems. In particular, growth-oriented values, such as self-transcendence and openness to change, as conceptualized in Schwartz’s value theory [11], emphasize intrinsic motivation, self-development, and engagement with higher-order goals. These values have been positively associated with both the presence of meaning and search for meaning, suggesting that individuals who endorse them are more likely to experience their lives as meaningful and to engage in efforts to deepen this meaning [14]. In turn, individuals reporting a greater sense of meaning in life tend to experience more positive effect, higher life satisfaction, and lower levels of psychological distress [7]. Although the search for meaning has often been linked to reduced well-being, it may reflect a developmentally appropriate form of engagement during periods of identity exploration, such as emerging adulthood [15]. Together, these findings support a conceptual framework in which meaning in life serves as a psychological mechanism linking personal values and psychological well-being.

**Figure 1:**
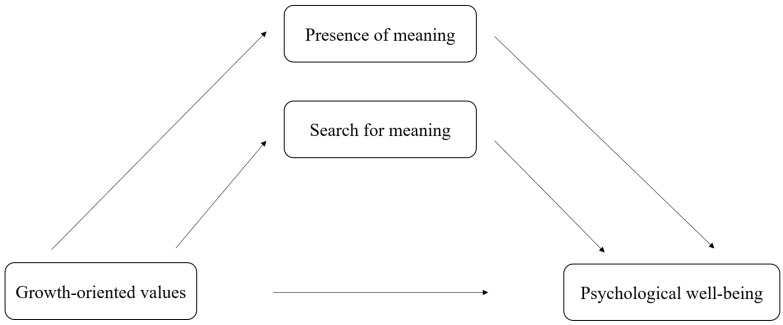
A conceptual framework of indirect effects involving meaning in life in the associations between growth-oriented values and psychological well-being.

The main aim of the study was to develop and empirically examine a conceptual model of the associations between psychological well-being, growth-oriented values, and meaning in life among emerging adults (Figure 1). Specifically, the study examined whether growth-oriented values are positively associated with psychological well-being (H1) and meaning in life (H2), and whether both the presence of meaning and the search for meaning are positively related to psychological well-being (H3). Finally, it was hypothesized that the presence of meaning and search for meaning might serve as potential indirect pathways linking growth-oriented values and psychological well-being (H4). Given the cross-sectional nature of the data, the study does not allow for causal inferences, and the analyses of indirect effects should be interpreted as exploratory and theoretically informed.

## 2. Materials and Methods

### 2.1. Participants

The sample consisted of 200 Polish emerging adults, 74% of whom were women and 26% men. The age range of the participants was 18–29 years (M = 21.90; SD = 2.48). The group was diverse in terms of education: 66% had completed secondary education, 27% held higher education degrees, 6.5% had primary education, and 0.5% vocational education. Regarding place of residence, 44.5% lived in cities with 150,000–500,000 inhabitants, 19% in rural areas, 15.5% in cities over 500,000, 14% in towns up to 50,000, and 7% in cities with 50,000–150,000 residents. In terms of living arrangements, 52% lived with parents or extended family, 23.5% with a partner, 13% with friends, and 11.5% lived alone. Occupational status varied: 39.5% were studying only, 38% were studying and working, 13.5% were in school but not at university, 7% were working only, and 2% were neither working nor studying. As for relationship status, 51% were single, 45% were in informal relationships, and 4% in formal ones. The data were collected in different parts of Poland. All 200 participants provided complete data, and no cases were excluded due to missing responses.

### 2.2. Procedure

The study was based on an online survey. The questionnaire link was shared on Facebook groups that bring together people in early adulthood. The inclusion criteria were ages 18 to 29, corresponding to the period of “emerging adulthood”. There were no additional exclusion criteria beyond age and informed consent. A convenience sampling strategy was used. The study had a cross-sectional design. To minimize potential biases, the survey was designed to ensure anonymity and voluntary participation, and recruitment targeted diverse online groups within the emerging adult population. The respondents were informed about the purpose and course of the study, the estimated duration, the possibility of withdrawing at any time, as well as the anonymity and confidentiality of individual results. All respondents gave their informed consent to participate in the study. Data collection took place during September and October 2024. Participants did not receive any financial reimbursement for their involvement. The project approval for the current study was obtained from The University Research Ethics Committee of the Institute of Psychology at the University of Szczecin (No. 41/2024, 17 October 2024) and was conducted according to the standards of the Declaration of Helsinki.

### 2.3. Measures

#### 2.3.1. Psychological Well-Being Scales

The Psychological Well-Being Scales (PWBS) is a self-report instrument designed to measure eudaimonic psychological well-being and its six dimensions. The scale was originally developed by Ryff [5] and has been adapted to Polish conditions by several researchers, including Krok [17]. The Polish short version consists of 42 items, grouped into six subscales: (1) *autonomy* (e.g., “My decisions are not usually influenced by what everyone else is doing”), (2) *environmental mastery* (e.g., “In general, I feel I am in charge of the situation in which I live”), (3) *personal growth* (e.g., “I have the sense that I have developed a lot as a person over time”), (4) *positive relations with others* (e.g., “Most people see me as loving and affectionate”), (5) *purpose in life* (e.g., “I have a sense of direction and purpose in life”) and (6) *self-acceptance* (e.g., “In general, I feel confident and positive about myself”). Participants rate each of the 42 statements using a single-choice response on a seven-point Likert scale ranging from 1 (strongly disagree) to 7 (strongly agree). Higher total scores indicate higher levels of psychological well-being. The reliability coefficient for the main scale used in the present study was Cronbach’s alpha = 0.94.

#### 2.3.2. Meaning in Life Questionnaire

The Meaning in Life Questionnaire (MLQ) is a self-report instrument designed to measure the sense of meaning in life. It was developed by Steger and colleagues [7], and adapted for Polish conditions by Kossakowska and colleagues [18]. The MLQ is a 10-item questionnaire that assesses two dimensions: (1) *presence of meaning* (the extent to which individuals feel their lives have meaning) (e.g., “My life has a clear sense of purpose”) and (2) *search for meaning* (the extent to which individuals are actively seeking meaning, purpose, and understanding) (e.g., “I am seeking a purpose or mission for my life”). Participants rate each of the 10 statements using a single-choice response on a seven-point Likert scale ranging from 1 (absolutely untrue) to 7 (absolutely true). Higher total scores indicate a greater sense of meaning in life. The reliability coefficients for the present study were Cronbach’s alpha = 0.89 for the presence of meaning and Cronbach’s alpha = 0.83 for the search for meaning.

#### 2.3.3. Portrait Values Questionnaire

The Portrait Values Questionnaire (PVQ-RR) is a self-report instrument designed to assess preferred personal values. It was developed by Schwartz and colleagues [11] and adapted for Polish conditions by Cieciuch and Schwartz [19]. The Polish version consists of 57 items, representing 19 narrowly defined value subscales, which are further grouped into four higher-order values: (1) *openness to change* (including: self-direction—thought, self-direction—action, stimulation, hedonism), (2) *self-transcendence* (including: benevolence—caring, benevolence—dependability, universalism—concern, universalism—nature, universalism—tolerance), (3) *self-enhancement* (including: achievement, power—dominance, power—resources, face), (4) *conservation* (including: personal security, societal security, conformity—rules, conformity—interpersonal, tradition, humility). Participants rate each of the 57 statements (descriptions of a person) by comparing them to themselves, using a single-choice response on a six-point Likert scale ranging from 1 (not like me at all) to 6 (very much like me). The scale does not yield an overall total score. The reliability coefficients for the present study were Cronbach’s alpha = 0.88 for openness to change, Cronbach’s alpha = 0.90 for self-transcendence, Cronbach’s alpha = 0.81 for self-enhancement, and Cronbach’s alpha = 0.85 for conservation.

### 2.4. Data Analysis

First, an a priori power analysis was conducted using G*Power 3.1.9.7 [20] to estimate the required sample size for the main analyses. The test specified was a linear multiple regression: fixed model, R^2^ increase. The results indicated that a minimum of 107 participants were needed to detect a medium effect size (f^2^ = 0.15), assuming a significance level of α = 0.05 and statistical power (1 − β) = 0.95. Ultimately, a larger sample (n = 200) was collected to enhance representativeness within the population of Polish emerging adults and to reduce the potential error in estimating effect sizes.

To assess the influence of individual observations on the regression models, three diagnostic measures were examined: Cook’s distance, Mahalanobis distance, and leverage (centered influence value). The results suggest that although some cases were relatively distant in the predictor space (particularly in Model 1), none exerted undue influence on the model estimates. Therefore, both regression models appear robust against the influence of individual data points, with no indication of distortion caused by outliers

The normality of the study variables, including psychological well-being, presence of meaning, search for meaning, openness to change, self-transcendence, self-enhancement, and conservation, was assessed using skewness and kurtosis values. All variables showed skewness and kurtosis coefficients within the acceptable range of ±1, suggesting no substantial deviations from normal distribution. All continuous variables were analyzed in their original form. No categorization of quantitative variables was applied.

Multicollinearity among predictors was evaluated using tolerance values and variance inflation factors (VIF). In both regression models, all tolerance values were above 0.7 and VIF values ranged from 1.18 and 1.27. These results fall well within the generally recommended thresholds, indicating no concerns regarding multicollinearity.

A hierarchical regression analysis was conducted to assess whether selected sociodemographic variables (sex, age, education level, place of residence, living arrangements, occupational status, and relationship status) acted as potential confounders in explaining psychological well-being. In the first step, these variables jointly accounted for approximately 8.8% of the variance (R^2^ = 0.088), with education level (β = 0.189, *p* = 0.046) and living arrangements (β = 0.154, *p* = 0.042) reaching statistical significance.

In the second step, psychological variables were included: presence of meaning in life, search for meaning, and either openness to change (Model 1) or self-transcendence (Model 2). The explained variance increased substantially (Model 1: R^2^ = 0.617; Model 2: R^2^ = 0.586). After adding these variables, none of the sociodemographic predictors remained statistically significant, suggesting that they did not exert notable confounding effects. Thus, the associations between psychological variables and well-being can be interpreted as relatively independent of the included sociodemographic characteristics.

Next, the following analyses were conducted using IBM SPSS Statistics, Version 28: (1) two-tailed correlations to examine initial associations among the study variables; (2) analyses of indirect effects to explore whether the presence of meaning and the search for meaning statistically account for the associations between openness to change and psychological well-being, and between self-transcendence and psychological well-being. As Hayes [21] recommended, Model 4 with bootstrapping was used (5000 samples; 95% bias-corrected confidence intervals).

## 3. Results

### 3.1. Initial Correlations Among Variables

First, Pearson correlation coefficients were used to examine the relationships among the study variables: psychological well-being, presence of meaning, search for meaning, openness to change, self-transcendence, self-enhancement, and conservation. Descriptive statistics, including means and standard deviations, were calculated for all study variables. The results are presented in Table 2.

Most variables were significantly correlated. Psychological well-being showed positive associations with the presence of meaning and the search for meaning, as well as with openness to change and self-transcendence. The presence of meaning was also related to the search for meaning, openness to change, and self-transcendence. Additionally, the search for meaning was associated with all other psychological constructs except self-enhancement. The strength of these relationships ranged from weak to strong.

### 3.2. Analysis of Direct and Indirect Effects

To explore whether the presence of meaning and search for meaning may account for the associations between openness to change and self-transcendence with psychological well-being, two separate models of indirect effects were tested using bootstrapping procedures (model 4, 5000 samples; 95% bias-corrected confidence intervals) [21]. In the first model, openness to change served as the predictor variable; in the second, self-transcendence. In both models, psychological well-being was the outcome variable, and the presence of meaning and the search for meaning were specified as intervening variables. Given the cross-sectional nature of the data, these analyses do not allow for causal inference and should be interpreted as theoretically guided, exploratory assessments of potential indirect associations. The results are presented in Table 3.

The regression model for openness to change reached statistical significance, F(3, 196) = 97.46, *p* < 0.001, and explained approximately 60% of the variance in psychological well-being (R^2^ = 0.60). Similarly, the model for self-transcendence was also significant, F(3, 196) = 82.76, *p* < 0.001, accounting for approximately 56% of the variance in psychological well-being (R^2^ = 0.56). Standardized beta coefficients (β) are reported alongside unstandardized 95% confidence intervals to provide both effect size and estimation precision.

For openness to change as an independent variable and psychological well-being as a dependent variable, openness to change was positively associated with both the presence of meaning (β = 0.32, 95% CI [0.15, 0.36]) and the search for meaning (β = 0.39, 95% CI [0.16, 0.32]). Among the two meaning-related variables, only the presence of meaning was a significant predictor of psychological well-being (β = 0.68, 95% CI [2.87, 3.82]). In contrast, the search for meaning did not show a significant effect on psychological well-being (β = –0.03, 95% CI [–0.86, 0.43]).

As a result, the indirect effect of openness to change on psychological well-being through the presence of meaning was significant (β = 0.22, 95% CI [0.50, 1.26]), whereas the indirect effect through the search for meaning was not (β = –0.01, 95% CI [–0.24, 0.15]). The total indirect effect was statistically significant (β = 0.21, 95% CI [0.42, 1.24]), indicating that only the presence of meaning may be interpreted as a significant intervening variable in this association. Since the direct effect of openness to change on psychological well-being remained significant after including both variables (β = 0.23, 95% CI [0.51, 1.29]), this pattern is consistent with partial mediation, although causal interpretations are not warranted due to the cross-sectional design.

A similar pattern was observed for the model in which self-transcendence served as the independent variable. Self-transcendence was positively related to both the presence of meaning (β = 0.28, 95% CI [0.09, 0.26]) and the search for meaning (β = 0.36, 95% CI [0.11, 0.24]). Again, only the presence of meaning significantly predicted psychological well-being (β = 0.72, 95% CI [3.03, 4.03]), while the search for meaning remained a non-significant predictor (β = 0.02, 95% CI [–0.53, 0.80]).

Accordingly, the indirect effect of self-transcendence on psychological well-being through the presence of meaning was statistically significant (β = 0.20, 95% CI [0.36, 0.91]), whereas the indirect effect via the search for meaning was not (β = 0.01, 95% CI [–0.13, 0.19]). Thus, similar to the first model, only the presence of meaning may act as a significant intervening variable in the association between self-transcendence and psychological well-being. The total indirect effect was significant (β = 0.21, 95% CI [0.35, 0.98]). Because the direct effect of self-transcendence on psychological well-being became statistically non-significant after accounting for both variables (β = 0.06, 95% CI [–0.12, 0.52]), this pattern is consistent with full mediation, though no causal conclusions can be drawn.

Overall, these findings suggest that the presence of meaning may help explain the observed associations between both openness to change and self-transcendence with psychological well-being, while the search for meaning does not. These results are consistent with partial mediation for openness to change and full mediation for self-transcendence, interpreted within the limitations of a cross-sectional design.

## 4. Discussion

The following discussion is grounded in the theoretical frameworks presented earlier, particularly Schwartz’s theory of basic human values [11], Steger’s conceptualization of meaning in life [7], and Ryff’s model of psychological well-being [5], which jointly provide the foundation for interpreting the observed results in the context of emerging adulthood.

This study investigated the relationships between psychological well-being, growth-oriented values, and meaning in life within a structural model tested among emerging adults. The findings were consistent with the assumed associations between growth-oriented values and psychological well-being, and indicated that the presence of meaning, but not the search for meaning, may help account for these relationships. To the best of our knowledge, this is one of the first empirical studies to examine how both dimensions of meaning in life are associated with the link between growth-related personal values and psychological functioning in emerging adulthood. As such, the study expands existing theoretical frameworks and contributes to a deeper understanding of the factors potentially linking personal values, meaning in life, and psychological well-being during this critical developmental stage.

### 4.1. Explaining Associations Between Psychological Well-Being and Personal Values

The results of the present study support the hypothesis (H1) that growth-oriented values are positively associated with psychological well-being among emerging adults. Participants who prioritize values linked to openness to change, such as personal development, the pursuit of new experiences, and openness to innovation and diversity, tend to report higher levels of psychological functioning. This includes dimensions such as self-acceptance, personal growth, autonomy, environmental mastery, purpose in life, and positive relations with others [5]. Similarly, individuals who emphasize self-transcendence values, which reflect care for others, social justice, and engagement in the common good or sustainable development, also show higher levels of psychological well-being. These findings align with previous research indicating that values directed toward personal growth and concern for others contribute to optimal mental functioning [22,23].

These results can be interpreted within the framework proposed by Sagiv and colleagues [24], who identified three theoretical perspectives explaining the relationship between values and well-being.

The first, *the healthy values perspective*, focuses on the content of values and assumes that certain values, particularly those that are intrinsic and growth-oriented, inherently promote well-being, while others may hinder it. Values such as self-direction, stimulation, and universalism, associated respectively with autonomy, novelty seeking, and concern for the broader social world, are typically positively related to psychological well-being. In contrast, values such as power and conformity, which emphasize status, control, or adherence to social norms, tend to correlate negatively with well-being outcomes [24,25]. This perspective draws on self-determination theory [26,27], which posits that intrinsic values (those satisfying basic psychological needs for autonomy, competence, and relatedness) are essential for psychological growth and flourishing.

The second, *the goal attainment perspective*, shifts the focus from what people value to how successfully they are able to pursue and realize their personally important goals. According to this approach, well-being increases when people are able to fulfill their goals and values, regardless of specific content, provided those values are meaningful and integrated into their sense of self [24]. This may help explain why values related to openness to change and self-transcendence, which tend to be intrinsically motivated and personally significant, are more strongly linked to well-being.

Finally, *the value congruency perspective*, emphasizes the role of social context, proposing that well-being is influenced by the alignment between a person’s individual value system and the dominant values in their surrounding environment. This congruence can support well-being by facilitating goal attainment, increasing social support, and reducing internal conflict [25]. For instance, Sortheix and Lönnqvist [28] demonstrated that congruence between personal and societal values predicts greater life satisfaction, particularly in societies that emphasize self-transcendence or openness to change.

Taken together, the current study not only corroborates earlier theoretical claims but also offers empirical evidence suggesting that growth-oriented values, both in terms of openness to change and self-transcendence, may be meaningfully associated with psychological well-being during the life stage of emerging adulthood.

### 4.2. Role of Meaning in Life in Associations Between Personal Values and Psychological Well-Being

The results support the hypothesis (H2) of a statistically significant relationship between both the presence of meaning and search for meaning in life and preferred growth-oriented values among emerging adults. Specifically, individuals who prioritize values associated with openness to change (such as striving for personal development, seeking new experiences, and being open to innovation and diversity) tend to report both a high presence of meaning in life and an active search for it. Likewise, individuals who endorse self-transcendence values (focused on caring for others, promoting social justice, and acting for the common good and sustainable development) are more likely to perceive their lives as meaningful, purposeful, and valuable, and to engage in efforts to deepen and better understand that meaning.

These patterns are consistent with findings by Besika and colleagues [29], who showed that individuals with a stronger orientation toward universal values reported higher levels of meaning in life. This finding also aligns with Schwartz’s theory [30], which emphasizes that values serve as interpretive frameworks that help individuals assign meaning to their experiences. As culturally embedded beliefs and motivational goals, values contribute to a sense of coherence and purpose, both of which are core components of meaning in life. According to Schwartz, when people’s actions are congruent with their personal values, they are more likely to experience their lives as meaningful.

The analyses also revealed statistically significant associations between psychological well-being, understood as positive psychological functioning across dimensions such as self-acceptance, personal growth, purpose in life, autonomy, environmental mastery, positive relations with others, and both dimensions of meaning in life: the presence of meaning, referring to the perception that one’s life is purposeful and meaningful and the search for meaning, understood as an active engagement in seeking or deepening that meaning (H3). This finding is consistent with previous research [31,32,33]. It is not unexpected, as Ryff herself emphasized, that meaning is central to the eudaimonic conceptualization of well-being, which underlies the theoretical framework adopted in this study [5].

Further analyses indicated that the presence of meaning in life, more than the search for meaning, plays a significant role in the associations between growth-oriented values and psychological well-being (H4). Specifically, in the case of openness to change, the presence of meaning was linked to psychological well-being alongside the direct association between values and psychological well-being. In contrast, for self-transcendence the association with well-being was primarily accounted for by the presence of meaning.

This suggests that individuals who prioritize openness to change values (such as self-direction—thought, self-direction—action, stimulation, and hedonism) tend to report higher psychological well-being, and part of this pattern may relate to their greater tendency to experience a clear sense of meaning and direction in life. Importantly, even when including the presence of meaning in the model, the association between openness to change values and psychological well-being remained significant. This implies that, beyond meaning in life, other psychological factors may also be associated with this relationship.

On the other hand, individuals who endorse self-transcendence values (such as benevolence—caring, benevolence—dependability, and various forms of universalism: social concern, environmental protection, and tolerance) tend to experience greater psychological well-being, largely in conjunction with experiencing a strong sense of meaning. In this case, the presence of meaning appears to be a particularly salient factor in understanding this link, suggesting that the positive association between self-transcendence values and psychological well-being may be stronger when these values are experienced as part of a coherent life purpose.

The observed difference between the roles of the presence of meaning and the search for meaning can be understood in light of existing theoretical and empirical work on the distinct psychological functions of these two dimensions. The presence of meaning reflects a stable sense that one’s life is coherent, purposeful, and significant [7]. As such, it is consistently associated with psychological well-being (particularly eudaimonic functioning) as it fulfills core existential needs for structure and direction [34].

In contrast, the search for meaning denotes a motivational tendency to seek or deepen one’s understanding of life’s significance. While it can serve as a positive force for personal growth and transformation, especially when accompanied by the presence of meaning, it may also emerge during periods of uncertainty or transition, when a sense of clarity has not yet been achieved [16]. As such, its association with well-being is more complex and context-dependent.

The current findings support this distinction, suggesting that growth-oriented values are more strongly associated with psychological well-being when they coexist with an already-established sense of meaning. In contrast, the ongoing search for meaning may reflect an exploratory process that, in itself, may not provide sufficient psychological integration to support well-being.

### 4.3. Limitations and Future Implications

While this study provides valuable insights into the areas of personal values, meaning in life, and psychological well-being, there are some limitations that should be acknowledged. One of the main limitations is its cross-sectional, correlational design, which does not allow for conclusions about directionality or causal relationships between variables. Therefore, the findings regarding indirect effects should be interpreted as exploratory. Future longitudinal research is recommended to verify the observed associations. Second, reliance on self-report measures may have introduced social desirability bias. The online nature of data collection also reduced experimental control and may have led to uncontrolled distractions. The sample size was relatively small (N = 200) and predominantly female (74%), which limits the generalizability of the findings. Additionally, values were analyzed using aggregated higher-order categories, which, while consistent with Schwartz’s model [11], may have obscured more nuanced value-specific effects.

Future research should consider including participants from other age groups, conducting longitudinal studies, and analyzing distinct components of psychological well-being and individual value types. Such approaches may enhance the precision and interpretive value of future findings.

These results may inform the design of preventative and educational programs that combine value clarification exercises with interventions focused on cultivating meaning in life. One promising direction involves encouraging self-transcendent goals that go beyond personal needs and promote concern for others, in line with Frankl’s theory of self-transcendence [35]. Another strategy is to facilitate meaning-making by helping individuals reinterpret life experiences and align personal values with long-term goals [36]. Such approaches may also be integrated into career counseling to support young adults in making life choices consistent with their deeper motivations and psychological well-being.

## 5. Conclusions

The current findings indicate that growth-oriented values, particularly openness to change and self-transcendence, are positively associated with psychological well-being among emerging adults. These associations appear to be especially strong when individuals report a high presence of meaning in life. Although the analyses explored potential indirect pathways involving meaning in life, the cross-sectional nature of the study does not permit conclusions about causal or temporal relationships. Therefore, the observed patterns should be interpreted as preliminary and exploratory. Nonetheless, the results underscore the relevance of meaning-making processes in the context of personal values and psychological functioning. Supporting the development of value orientations and fostering a sense of meaning may be beneficial for psychological well-being during the transition to adulthood, but further longitudinal research is needed to clarify these relationships.

## Figures and Tables

**Table 1:** Schwartz’s 19 values and their classification into four higher-order value groups.

	Growth	Self-Protection
Personal focus	*Openness to change* Self-direction—thought Self-direction—action Stimulation Hedonism	*Self-enhancement* Achievement Power—dominance Power—resources Face
Social focus	*Self-transcendence* Benevolence—dependability Benevolence—caring Universalism—concern Universalism—nature Universalism—tolerance	*Conservation* Security—personal Security—societal Tradition Conformity—rules Conformity—interpersonal Humility

**Table 2:** Descriptive statistics and correlations among psychological well-being, presence of meaning, and search for meaning, and groups of personal values in emerging adults (N = 200).

Variables	M	SD	Psychological Well-Being	Presence of Meaning	Search for Meaning
1. Psychological well-being	200.20	37.21	-		
2. Presence of meaning	22.24	7.59	0.74 **	-	
3. Search for meaning	25.49	5.82	0.30 **	0.35 **	-
4. Openness to change	55.58	9.55	0.44 **	0.32 **	0.39 **
5. Self-transcendence	71.22	11.91	0.27 **	0.28 **	0.36 **
6. Self-enhancement	45.24	9.07	0.08 (*p* = 0.26)	0.11 (*p* = 0.12)	0.11 (*p* = 0.13)
7. Conservation	71.62	13.68	0.10 (*p* = 0.16)	0.17 (*p* = 0.02)	0.26 **

** *p* < 0.01.

**Table 3:** Direct and indirect associations between growth-oriented values and psychological well-being with presence of meaning and search for meaning as potential intervening variables (N = 200).

Pathways	β	95% CI		SE	t
		LL	UL		
**Model 1**					
*Direct effects*					
Openness to change ⭢ Presence of meaning	0.32 ***	0.15	0.36	0.05	4.76
Openness to change ⭢ Search for meaning	0.39 ***	0.16	0.32	0.04	6.00
Presence of meaning ⭢ Psychological well-being	0.68 ***	2.87	3.82	0.24	13.78
Search for meaning ⭢ Psychological well-being	−0.03	−0.86	0.43	0.33	−0.66
Openness to change ⭢ Psychological well-being	0.23 ***	0.51	1.29	0.20	4.60
*Indirect effects*					
Indirect total effect	0.21	0.42	1.24	0.21	-
Openness to change ⭢ Presence of meaning ⭢ Well-being	0.22	0.50	1.26	0.19	-
Openness to change ⭢ Search for meaning ⭢ Well-being	−0.01	−0.24	0.15	0.10	-
*Total effect*					
Openness to change ⭢ Psychological well-being	0.44 ***	1.21	2.19	0.25	6.83
**Model 2**					
*Direct effects*					
Self-transcendence ⭢ Presence of meaning	0.28 ***	0.09	0.26	0.04	4.08
Self-transcendence ⭢ Search for meaning	0.36 ***	0.11	0.24	0.03	5.47
Presence of meaning ⭢ Psychological well-being	0.72 ***	3.03	4.03	0.25	13.97
Search for meaning ⭢ Psychological well-being	0.02	−0.53	0.80	0.34	0.40
Self-transcendence ⭢ Psychological well-being	0.06	−0.12	0.52	0.16	1.23
*Indirect effects*					
Indirect total effect	0.21	0.35	0.98	0.16	-
Self-transcendence ⭢ Presence of meaning ⭢ Well-being	0.20	0.36	0.91	0.14	-
Self-transcendence ⭢ Search for meaning ⭢ Well-being	0.01	−0.13	0.19	0.08	-
Total effect					
Self-transcendence ⭢ Psychological well-being	0.27 ***	0.43	1.27	0.21	3.97

Note. Standardized coefficients (β) are reported. 95% CI = 95% confidence interval for the unstandardized coefficient; SE = standard error; t = t-value. *** *p* < 0.001

## Data Availability

The raw data supporting the conclusions of this article will be made available by the authors on request due to its sensitive ethical nature.
